# A New Method of Removing Foreign Bodies from the Joints

**Published:** 1843-10

**Authors:** G. Goyrand

**Affiliations:** Chief Surgeon to the Hospital at Aix; Corresponding member of the Royal Academy of Medecine, &c.


Art. II.—*A New Method of removing Foreign Bodies from the Joints.* By G. Goyrand, M.D., Chief Surgeon to the Hospital at Aix; Corresponding member of the Royal Academy of Medecine, &c. Translated from the French for the Western Journal of Medicine and Surgery, by E. L. Grant, M.D., of Louisville.

The generalization of the method of subcutaneous incision is a great improvement in surgery. Every day new applications of this method may be made; it renders exempt from all danger operations which, performed by opening the joint, are very severe and often fatal.

Foreign bodies in the articulations render motion painful, and constitute a serious infirmity, particularly when situated in the knee. Should they be extracted? We know the danger of penetrating wounds of the articulations; and if we consult authors on the results of the extraction of these substances, how often shall we find that this operation has been followed by the most fearful consequences! Richerand performed it twelve times; four of the patients died, and of those who recovered, one, a young girl, was cured with great difficulty. Dupuytren moved a foreign body from the tibio-tarsal articulation: the wound being somewhat irritated in consequence of the concretion not being steadied by the fingers of the operator, fatal arthritis supervened.* In November, 1835,

*Bégin, Leçons orales au Val-de-Grâce. Octobre 5, 1840.

Lisfranc, extracted a foreign body from the knee of a healthy, robust, young man: the patient died. In 1839, the same surgeon again performed the operation, and with a similar result.*

In May, 1839, a man 35 years of age, was admitted at the Hôtel-Dieu of Nantes, who had in his right knee a very moveable concretion. This substance was extracted without difficulty, the patient survived the operation but a few days.†

Roux performed a similar operation on the right knee of a young man, who had been received into the Hôtel-Dieu, for orchitis; a violent arthritis supervened, followed by excessive suppuration, which rendered it necessary to amputate at the thigh; the patient died soon after. Finally, this operation, performed October 5th, 1840, by Bégin, at Val-de-Grâce, was likewise followed by death.‡

We have here cited a number of cases in which the removal of foreign bodies from the articulations caused death. We are generally more willing to publish our success than our failures; nevertheless, we should in vain seek in the periodical collections of late years an equal number of cases in which this operation resulted favorably. When, after the operation, the synovial membrane becomes inflamed, should the patient be so fortunate as to escape death, which unhappily is very seldom the case, he is ever after troubled with anchylosis, as has been proved by the observations of Richerand, Kirby, and Boyer.

With the knowledge of these dangers, surgeons have hesitated to perform this operation, and have sought to render it unnecessary by so placing the substance that it may cause less inconvenience. With this view, some, among whom are Gooch, Middleton, Hays, and Boyer, have suggested the method of pushing it into a cul-de-sac of the synovial membrane, at a distance from the centre of the joint, and keeping

---

* Gazette des Hôpitaux, 31 Août, 1839, et 27 Octobre, 1840.

† Journal de la Section de Médicine de la Sociéte Académique de la Loire-Inférieure, 67e Livraison.

‡ Gazette des Hôpitaux, 27 Octobre, 1840.

it in this position by means of a tight bandage, a laced stocking, or a knee cap, in the hope that it will fix itself there by contracting adhesions. Others have thought it best to produce anchylosis by protracted immobility of the limb. B. Bell considers the extraction of these bodies so dangerous, that in cases in which they are adherent, and where consequently their removal would present difficulties, requiring much search and handling of the part, he recommends amputation of the limb.*

But recent improvements in surgery have opened to us new means. The harmlessness, so well demonstrated by Julius Guérin, of subcutaneous wounds even when they penetrate the joints, has suggested to me the idea of a new operation, which will remove this infirmity without involving the least danger. This operation is particularly described in the following cases.

Case 1.—J. B. A., aged 24 years, came to the Hospital of Aix, September 14th. He had in his right knee a foreign body which was very annoying, frequently causing acute pain. This concretion, about the size of an almond, was free in the synovial membrane. On the first examination of the patient, the substance was on the outer side of the articulation. We pushed it above the patella, then to the internal side of this bone, and afterwards under the ligament of the patella, where it was lost. When thus hidden between the articular surfaces, it occasioned pain and swelling of the knee; and would generally reappear when the patient walked on an uneven surface.

On the 15th the concretion was found situated as on the first examination; the knee was slightly swollen and painful. Repose and cataplasms were ordered, and on the following day the swelling and pain had subsided. The patient was very desirous to be freed of his infirmity. Formerly I should not have consented to operate; because I do not think a surgeon ought to perform a dangerous operation, to cure a sim-

* Cours complet de Chirurgie, traduit par Bosquillon t. v. p. 282.

ple infirmity; but the idea struck me that I might in this case, make a new application of the method of subcutaneous incision. I thought to extract the foreign body at two operations. In the first, I would divide the synovial membrane over the foreign body, together with the muscular and fibrous tissues which cover it, and push the substance across the opening into the subcutaneous cellular tissue. In the second, performed ten or twelve days after, when the subcutaneous wound should have become perfectly cicatrized, I would extract the body by a simple incision. This idea pleased me; but I wished to reflect upon it for a few days.

The inquiry arose in my mind, would not these concretions, allowed to remain in the organised tissues, immediately produce suppurative inflammation, which might communicate itself to the articulation? I thought not; these bodies are neither very large, heavy, nor rough; being analagous in their texture to the living tissues; and are so situated that the air cannot penetrate to them. It seemed to me that these circumstances should prevent suppurative inflammation. At what point should the incision be made? The concretion might be moved into any of the situations of the articular cavity. At the sides of the knee, at the articular interline, or very near it, it is covered but by the skin, a fibrous layer, and the synovial membrane; but then I should not be able to fix it steadily. I decided therefore to push it to the upper part of the synovial membrane, into the cul-de-sac formed at this point by the reflexion over the articular surfaces. But then the question recurs, at what particular point of the cul-de-sac? There it would be necessary to make the incision through the anterior straight muscle of the thigh, its edges not being sufficiently separable to allow of the extraction of the concretion. On the inside of this muscle, the cul-de-sac of the synovial membrane is much nearer the articular interline than on the outer side. It would be better to make the incision through the vastus externus. At this point the concretion could be easily fixed by the fingers: the longitudinal incision would divide the vastus externus muscle obliquely, which would separate so as to allow of the passage of the foreign body. The bistoury I used was stout, narrow, sharp pointed, and fixed on a spring handle. I operated December 22d, in the following manner:

The patient being placed on a bed, I stood at his left-side. I pushed the foreign substance into the outer part of the superior cul-de-sac of the synovial membrane, a short distance above the patella, where I fixed it, continuing to press it from below upward, with the left thumb and index finger. An assistant then raised the skin of the thigh, above the concretion, in a large transverse fold; in this way I brought towards this body a portion of skin originally distant from it. I plunged my bistoury from above downwards, through the base of this fold, and directing the point towards the foreign substance, I cut under the skin, parallel with the axis of the limb, all the tissues that covered the body, which latter I found to be of the consistence of bone: I was obliged to make three cuts before I could divide the tissue, when I felt the extraneous body slip from under my fingers, and found that it had passed out of the articulation. I then withdrew my bistoury, and the assistant let go the fold of the skin. Some drops of blood mixed with bubbles of air escaped through the puncture of the skin, which was situated a short distance above the point where I had cut the synovial membrane. A few bubbles of air remained in the cellular tissue below the opening.

The foreign body had not passed under the skin, as I had wished, but glided between the middle and outer part of the triceps muscle. I left it a short distance above the incision of the synovial membrane.

I covered the wound, which was very small, with adhesive plaster. A thick compress was held by a circular band upon the point where the triceps muscle, aponeurosis, and the synovial membrane had been divided; with the double object of compressing the subcutaneous wound and preventing the retrocession of the concretion, which was above. Contrary to the directions that had been given, the patient left his bed three times on the day of the operation, notwithstanding which no serious consequences followed. The wound in the skin healed up in twenty-four hours. The plastic lymph, which filled the opening made by the bistoury, formed then a small tumour which, however, was by no means painful; there ensued in the joint neither pain nor stiffness.

I removed the compress and bandage six days after the operation, when I found in the articulation another foreign body the size of a pea, moveable like the first. Two days after, a third presented itself, very nearly the size of the one I had dislodged. This one concealed itself between the articular surfaces, and did not re-appear until the 8th of October; the patient fixed them both on the outside of the articulation, until I arrived. Whilst I examined the two concretions, the smaller one escaped from beneath my fingers and concealed itself under the patella. The larger one was hard, and from the sensation it imparted on friction against the osseous surfaces, I supposed it to be rough. I should have preferred dislodging both the substances by the same incision; but, fearing that the larger one should again become concealed, I decided on removing it immediately, and leaving the other, it being two small to occasion much inconvenience.

The synovial membrane irritated by the foreign body which had remained several days between the osseous surfaces, contained a large quantity of effused synovia. It would, perhaps, have been better to defer the operation, as on the former occasion, until after all irritation should have subsided; but the subcutaneous incision seemed to me so harmless that I did not fear to make it notwithstanding this circumstance. I was aware that on the division of the synovial membrane the fluid would escape, and pass into the cellular tissue, but would not be discoverable the day after the operation.

On the 8th of October, I fixed the foreign body in the upper cul-de-sac of the synovial membrane, a little nearer the median line than the first, but still outside of the straight muscle. The skin was raised as in the first operation, with a single stroke of the bistoury I divided the aponeurosis, triceps muscle, and the synovial membrane from below, upwards. In withdrawing the instrument, I was careful to divide sufficiently the aponeurosis and triceps muscle under the skin. As soon as the deep tissues were divided, the body slipped under my fingers and passed along the blade of the bistoury into the sub-aponeurotic cellular tissue. The subcutaneous cellular adipose stratum being very thin in this patient, the displaced concretion was very near the surface. I pushed it up a short distance above the opening in the synovial membrane, and left it there, on the external side of the wound in the skin. The wound was dressed as in the first operation, and the patient required to keep his bed. The results of this operation were as simple and as happy as those of the first. The day after, the opening in the skin was healed, the effusion of the synovia had entirely disappeared, and the next day A. walked about the Hospital without experiencing the slightest pain. The small concretion which remained in the knee caused no inconvenience.

After the lapse of eleven days, the foreign substances that I had removed from the articulation into the cellular tissue had caused neither constraint nor pain. Were I to leave them there they would become encysted; the first of these bodies had been one month in the place where I had left it; its cyst must be already formed. The patient might now be considered cured; but I wished to finish the operation as I had at first intended. October 19th.—I proceeded to extract the foreign body, which I had last dislodged. Holding it fixed with the left hand, I exposed it by a small incision, including the skin and aponeurosis—then seizing it with a pair of dissecting forceps, removed it. This body was solid and appeared already encysted; so that this second operation was evidently useless. I did not wish to extract the concretion that was under the vastus externus muscle, as this would unnecessarily complicate the operation. The foreign body which I had removed was of an irregular form, twenty-two millimetres in length, eighteen in width, and eleven millimetres at its greatest thickness. One of its sides smooth, regular, and very slightly convex, was cartilaginous; the other, irregular and knotted, was osseous. On sawing this substance it had the appearance, hardness, and density of ivory, and was covered on the smooth side with a regular cartilaginous layer nearly two millimetres in thickness, having the appearance of a diarthrodial cartilage. This body was situated in the cellular tissue, its cartilaginous surface towards the apponeurosis and the small extremity upwards. The edges of the incision were exactly approximated, and the patient again ordered to remain in bed. Notwithstanding these precautions the wound suppurated, and on the 3d November, it became necessary to re-open the wound to give issue to the pus collected under the aponeurosis. November 14th.—The wound was not yet entirely healed, and there was a dull pain in cicatrix, which, however, did not extend to the joint.

The removal of foreign bodies from under the skin, after they are dislodged, is not in any case a dangerous operation; but, in my opinion, an entirely useless one. Henceforth I shall allow them to remain in the cellular tissue.

Nearly all foreign bodies in the articulations may be extracted by my method. If instead of a hard body, we encounter a cartilaginous one, it will be divided by the bistoury, but the fragments may be as easily dislodged as entire concretions.

These substances are sometimes found attached to the wall of the synovial membrane, by a membraneous pedicle, which may be easily divided. The operation will then consist, first, in cutting the body from its attachment, and then, after the lapse of several days, dislodging it.

Should several foreign bodies be found in the joint at the same time, the incision should not be made until we have brought them all to one point, so as to allow them to pass through the same opening. I should have observed this precaution in A's case, had I suspected the existence of two concretions. Perhaps I ought to have deferred my second operation until the small body which remained in the knee could be dislodged with the large one; but I feared this latter might again disappear between the osseous surfaces; and besides, the other was so small that it could not materially incommode the patient. Should it increase, I will remove it as I did the other two.

I have several times extracted foreign substances from the articulation, on dead subjects. On one occasion, I removed the scaphoid bone of the carpus, after having introduced it into the articular cavity of the knee, by a small opening made at the side of the patella. In none of these experiments did the foreign body pass between the middle and the outer fascia of the triceps muscle, as in A's case; but it invariably lodged itself in the sub-aponeurotic cellular tissue, never under the skin. The subcutaneous cellular adipose tissue is too dense to allow this body to penetrate it. It is of little consequence to the success of this operation whether the dislodged body passes between the fascia of the triceps muscle, or in the sub-aponeurotic cellular tissue; as in either case it becomes encysted. In A's case, in which the dislodged body produced suppurative inflammation, it would have been better had the concretion been near the surface. We should endeavor to leave the foreign body under the aponeurosis; and we shall always be able to do so, if the vastus externus muscle be sufficiently incised.

I place entire confidence in the operation I have just described; it is simple and innocent, and is destined to displace one more dangerous than that for strangulated hernia.

